# Detailed characterization of antibody responses against HIV-1 group M consensus gp120 in rabbits

**DOI:** 10.1186/s12977-014-0125-5

**Published:** 2014-12-20

**Authors:** Yali Qin, Heliang Shi, Saikat Banerjee, Aditi Agrawal, Marisa Banasik, Michael W Cho

**Affiliations:** Department of Biomedical Sciences, College of Veterinary Medicine, Iowa State University, 1600 S 16th Street, Ames, IA 50011-1250 USA; Center for Advanced Host Defenses, Immunobiotics and Translational Comparative Medicine, Iowa State University, Ames, IA 50011 USA

**Keywords:** HIV-1 vaccine, Neutralizing antibody, eOD-GT6, gp120, SOSIP gp140, Immunogenic epitope, Antibody competition

## Abstract

**Background:**

We recently reported induction of broadly neutralizing antibodies (bnAbs) against multiple HIV-1 (human immunodeficiency virus type 1) isolates in rabbits, albeit weak against tier 2 viruses, using a monomeric gp120 derived from an M group consensus sequence (MCON6). To better understand the nature of the neutralizing activity, detailed characterization of immunological properties of the protein was performed. Immunogenic linear epitopes were identified during the course of immunization, and spatial distribution of these epitopes was determined. Subdomain antibody target analyses were done using the gp120 outer domain (gp120-OD) and eOD-GT6, a protein based on a heterologous sequence. In addition, refined epitope mapping analyses were done by competition assays using several nAbs with known epitopes.

**Results:**

Based on linear epitope mapping analyses, the V3 loop was most immunogenic, followed by C1 and C5 regions. The V1/V2 loop was surprisingly non-immunogenic. Many immunogenic epitopes were clustered together even when they were distantly separated in primary sequence, suggesting the presence of immunogenic hotspots on the protein surface. Although substantial antibody responses were directed against the outer domain, only about 0.1% of the antibodies bound eOD-GT6. Albeit weak, antibodies against peptides that corresponded to a part of the bnAb VRC01 binding site were detected. Although gp120-induced antibodies could not block VRC01 binding to eOD-GT6, they were able to inhibit VRC01 binding to both gp120 and trimeric BG505 SOSIP gp140. The immune sera also efficiently competed with CD4-IgG2, as well as nAbs 447-52D, PGT121 and PGT126, in binding to gp120.

**Conclusions:**

The results suggest that some antibodies that bind at or near known bnAb epitopes could be partly responsible for the breadth of neutralizing activity induced by gp120 in our study. Immunization strategies that enhance induction of these antibodies relative to others (*e.g.* V3 loop), and increase their affinity, could improve protective efficacy of an HIV-1 vaccine.

## Background

The human immunodeficiency virus type 1 (HIV-1) gp120 and gp41 envelope glycoproteins are the sole targets for neutralizing antibodies (nAbs). Extensive antigenic variation of the virus poses a major scientific challenge for developing an effective AIDS vaccine [1]. In particular, the inability to induce broadly neutralizing antibodies (bnAbs) against the large number of HIV-1 variants is the biggest obstacle. For an effective nAb response to be elicited by an HIV vaccine, this genetic variation must be overcome at the antigenic level. Using a centralized HIV-1 gene sequence as an immunogen is one strategy to overcome diversity challenge in HIV-1 vaccine development. Sequences generated by centralized methods include consensus, ancestral, mosaic, and phylogenetic tree center [2-7].

The M group consensus sequence MCON6 was first reported as a biologically functional immunogen by Gao *et al.* [8]. In this study, MCON6 env gp120 and gp140CF induced both T-cell immune responses (in BALB/c mice) and neutralizing antibodies against HIV-1 primary isolates (in guinea pigs). The neutralizing activities were weak and mostly induced towards the V3 loop. When using a DNA-prime-recombinant vaccinia virus boost, the MCON6-derived vaccine induced a greater number of T-cell epitope responses than any other tested single wild-type subtypes [9]. Later, a second M group consensus sequence (CONS) was published, which was based on a more comprehensive collection of HIV-1 env sequences and contained shorter variable loop sequences [10]. Santra *et al*. demonstrated in rhesus macaques that CONS based immunogens elicited cellular immune responses with significantly increased breadth compared to immunogens expressing a wild-type virus gene [11]. Liao *et al*. found that the best inducer of potent nAb responses was the CONS Env when studied as part of a panel of twenty recombinant transmitted/founder (T/F), chronic, and consensus gp140 envs [12]. However, subsequent studies demonstrated that MCON6 and MCONS are comparable immunogens, and there were no significant differences in humoral or T-cell immune responses [10,13]. Both M group consensus immunogens induced higher immune responses than wild type immunogens [8-10,13]. Additionally, both MCON6 and MCONS induced greater cross-reactive HIV-neutralizing antibodies as compared with wild-type immunogens [10].

We recently reported the comparison of the immunogenic properties of three MCON6 derived envelope proteins. These included a soluble, monomeric gp120 and two immunogens based on the outer domain (gp120-OD and gp120-ODx3). Our outer domain immunogens induced greater neutralizing antibody responses than others previously examined [14]. The full-length gp120 also elicited potent nAbs against multiple HIV-1 isolates from different clades, albeit largely against tier 1 viruses. Surprisingly, when compared to OD and ODx3, gp120 induced better nAbs against tier 2 pseudoviruses, albeit weak, when using a more sensitive assay based on A3R5 cells.

Currently, detailed information on humoral immune responses against M group consensus envelope immunogens is not available. Information on the kinetics of antibody induction against specific epitopes, the level and specificity of antibodies against conformational epitopes, and cross-reactivity to other envelope vaccine candidates could provide valuable information in designing better immunogens and formulating more effective vaccine strategies. As such, we carried out more detailed analyses to better characterize immunogenic properties of MCON6 gp120.

## Results

### Spacial-temporal analyses of immunogenic linear epitopes on gp120

In the previously published report [14], we identified immunogenic linear epitopes within the gp120 outer domain portion by conducting overlapping peptide ELISA with immune sera collected after the fifth immunization. To better characterize immunogenic properties of the protein, we extended epitope mapping analyses to identify immunogenic epitopes in the inner domain. Additionally, immune sera collected after the second and fourth immunizations were examined to assess kinetic parameters of B cell responses against the antigen.

**Figure 1:**
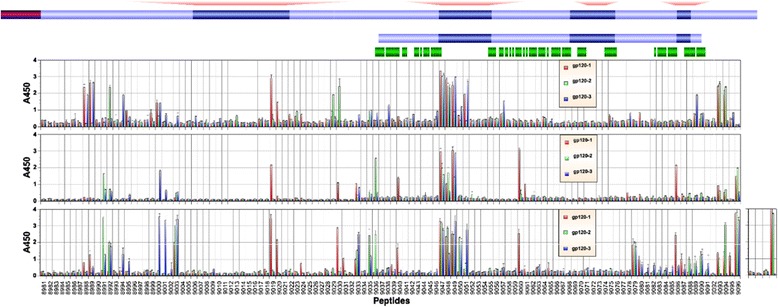
**Identification of immunogenic linear epitopes by ELISA using overlapping peptides.** ELISA was conducted with antisera collected from three rabbits after the second, fourth and fifth immunizations using overlapping peptides. Peptide numbers represent catalog numbers from the NIH AIDS Reagent Program. A schematic diagram of gp120, as well as gp120-OD, is shown on top. Corresponding regions on the eOD-GT6 construct and identical amino acids are shown in green bars. MCON-S sequence, from which peptides are derived, is shown. Amino acid residues that differ on MCON6, from which the immunogen is generated, are indicated with small red squares. Sequences of MCON6-specific peptides (V1, V2, V4 and V5) are indicated in red text at the top. Blue circles below the schematic diagram of gp120 indicate amino acid residues that make contact with CD4 and/or VRC01. A450 represents absorbance value at 450 nm.

As expected, the V3 loop was the most immunogenic region, inducing higher antibody levels in all three rabbits at all three time points analyzed (Figure 1). In particular, three peptides that cover the N-terminal half (9047, 9048, and 9049) were highly reactive in all three animals. The only other peptides that induced high titer antibodies in all animals (*viz*. A450 reading of greater than 1 at 1:100 dilution) were peptides 9003 (C1), 9033 (C2), 9079 (C4), and 9096 (C5). For all these latter peptides, antibody titers were low or undetectable after the second immunization. However, titers increased with each successive immunization. In particular, peptide 9096 (APTKAKRRVVEREKR), which is situated at the very C-terminal end of gp120, was the single most immunogenic peptide after the fifth immunization.

Although antibody responses generally increased with each booster immunization, there were some notable exceptions in which antibody levels declined substantially (*e.g*. peptides 8988 and 8989 for rabbits #1 and #3; peptides 9029 and 9030 for rabbit #2). Unsurprisingly, this was not consistently observed all rabbits; in contrast to rabbit #2, antibody titers against peptide 9030 increased in rabbit #1. There were other animal-to-animal variations in the immunogenicity of individual peptides. Peptides 9019, 9020, 9060 and 9086 were immunogenic only in rabbit #1; peptide 9036 was immunogenic only in rabbit #2; and peptides 9000 and 9001 were immunogenic only in rabbit #3.

Besides the V3 loop, the C1 and C5 regions were immunogenic, which is consistent with what was observed in mice immunized with gp160/gp120 (HIV-1_DH12_ strain) in a prime-boost immunization regimen [15]. However, in marked contrast to what was observed in mice, the V1/V2 region was quite non-immunogenic; only one animal mounted antibodies against peptides 9019 and 9020. Presently, we do not know whether this difference is from the difference in the immunogen source (MCON6 sequence *vs*. DH12 strain), immunization strategy (gp120 *vs*. gp160 rVV/gp120 prime-boost) and/or the difference in the animal species used (rabbit *vs*. mouse).

**Figure 2:**
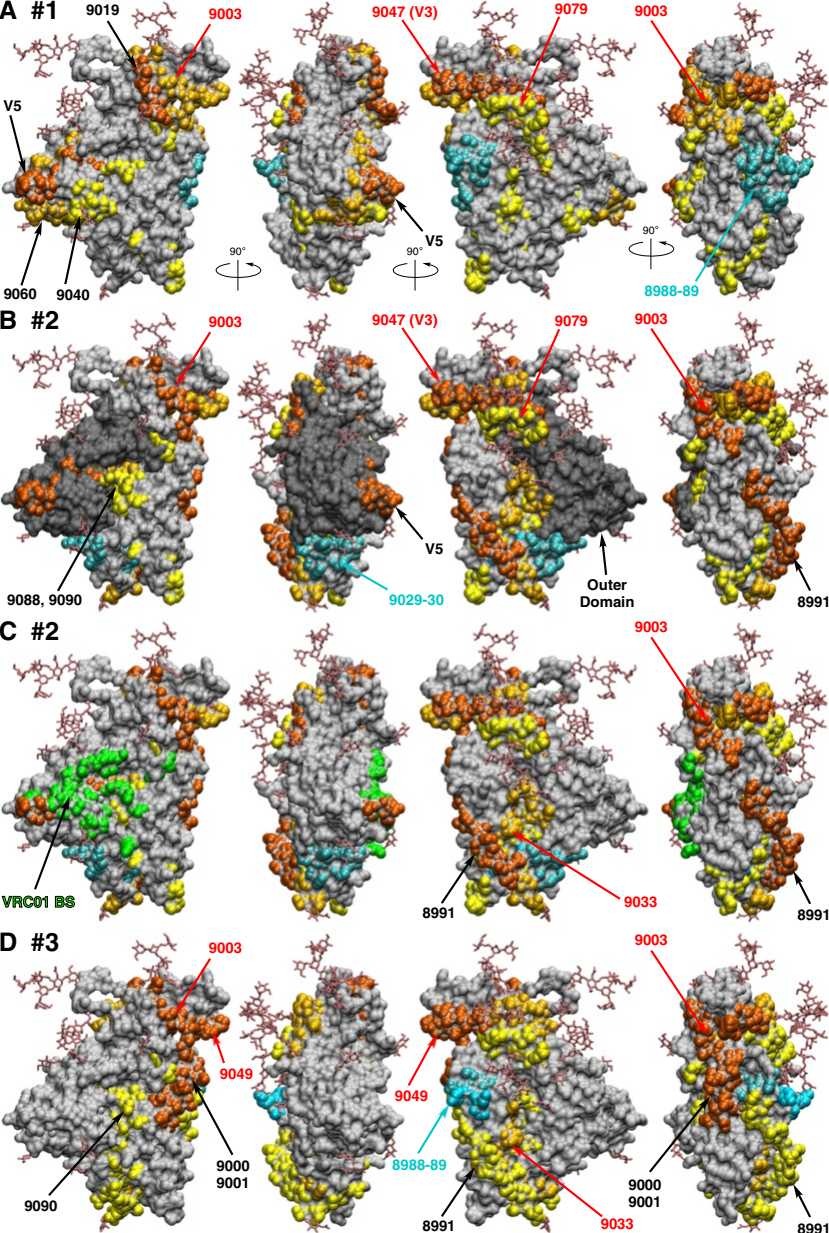
**Immunogenic linear epitopes plotted onto the surface of gp120.** ELISA data from Figure 1 were used to illustrate immunogenic epitopes for rabbits #1 **(A)**, #2 **(B and** **C)**, and #3 **(D)**. A450 reading between 1–2, 2–3, and 3–4 are shown in yellow, gold and orange, respectively. Peptides with reduced antibody reactivity after the second immunization are shown in cyan. Peptides that were reactive in all three animals are indicated in red text. Outer domain is shown in panel B in dark grey and VRC01 binding site is shown in green in panel C. The crystal structure of SOSIP gp140 (accession code 4NCO) was used to generate figures.

To better understand spatial distribution of immunogenic epitopes, regions corresponding to immunoreactive peptides were plotted onto a recently solved crystal structure of BG505 SOSIP gp140 [16]. Peptides are color-coded based on immunogenicity (yellow, gold and orange for A450 values between 1–2, 2–3 and 3–4 by ELISA, respectively) and plotted for each rabbit individually (Figure 2). Peptides with reduced antibody reactivity after the second immunization are shown in cyan. Many immunogenic epitopes were clustered together even when distantly separated in primary sequence. For example, in rabbit #1, peptide 9040 (in C2), peptide 9060 (in C3) and V5 loop are close together (Figure 2A). Similarly, peptides 9003 (C1), 9019 (V2), 9047–9 (V3) and 9079 (C4) form a contiguous ring of immunogenic epitopes on top of the envelope spike.

As shown in Figure 2B, the outer domain (shaded in dark grey) was mostly non-immunogenic, other than the V3 and V5 loops, and peptide 9079 in the C4 region became moderately immunogenic only after the fifth immunization. Interestingly, there were a few weakly immunoreactive peptides (peptides 9040, 9088 and 9090) that partly overlap amino acid residues that comprise the VRC01 binding site (indicated in green in Figure 2C). This raises the possibility that some antibodies that bound these peptides could interfere with binding of gp120 to CD4.

### Induction of antibodies directed against the gp120 outer domain

**Figure 3:**
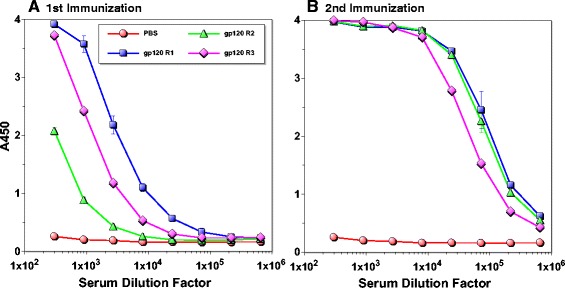
**Assessment of antibodies directed against gp120 outer domain.** ELISA was performed using rabbit immune sera after the first **(A)** and second **(B)** immunization using gp120-OD as the coating antigen. Serum samples from a mock-immunized animal are indicated as “PBS”.

Although linear epitope identification *via* overlapping peptide ELISA can provide insights into immunogenic properties of antigens, it provides only limited information. This is particularly a problem in assessing antibody responses against a region comprised of multiple, non-contiguous protein segments (*e.g.* CD4BS on gp120). To better understand immunogenic properties of regions critical for inducing bnAbs (*e.g.* VRC01), we first assessed antibody response levels against the entire gp120 outer domain. To do this, ELISA was performed with gp120-OD as the coating antigen (Figure 1, [14]). As shown in Figure 3A, fairly potent antibody responses were induced in two rabbits even after a single immunization. After the second immunization, all three animals induced strong antibody responses against the outer domain with end point titers greater than 1×10^6^ (Figure 3B).

Based on linear epitope mapping analyses, the vast majority of antibodies induced after the second immunization targeted the V3 loop (Figure 1). However, we were curious as to whether any non-V3 loop-antibodies that bound discontiguous, conserved epitopes were induced (*viz*. CD4BS). To quantify the level of these antibodies, we used the recently published eOD-GT6, an engineered outer domain that can efficiently bind multiple VRC01-class bnAbs and their germline precursors [17]. As shown in Figure 1, eOD-GT6 lacks the V3 loop and significant portions of V4 and V5 regions. It should be noted that there are multiple amino acid sequence variations between eOD-GT6 and our gp120 based on MCON6 sequence (74% identity, not including the linker sequences on eOD-GT6). Thus, eOD-GT6 is highly suited for measuring antibodies that may bind to conserved amino acid residues targeted by bnAbs.

**Figure 4:**
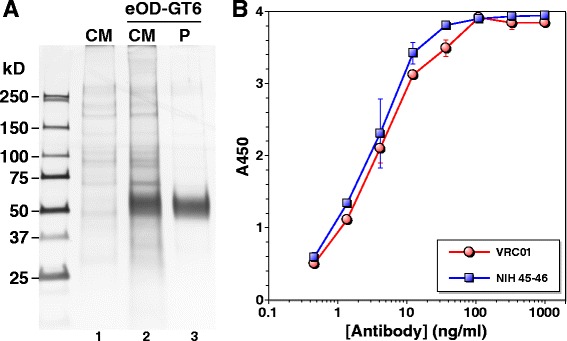
**Expression, purification and antigenic analysis of eOD-GT6. (A)** Expression and purification of eOD-GT6. Protein was detected by silver staining. Lane 1: culture medium (CM) of mock transfected cells; lane 2: CM of eOD-GT6 transfected cells, 4 days after transfection; lane 3: purified (P) eOD-GT6. **(B)** eOD-GT6 ELISA with VRC01 (red) and NIH45-46 (blue).

**Figure 5:**
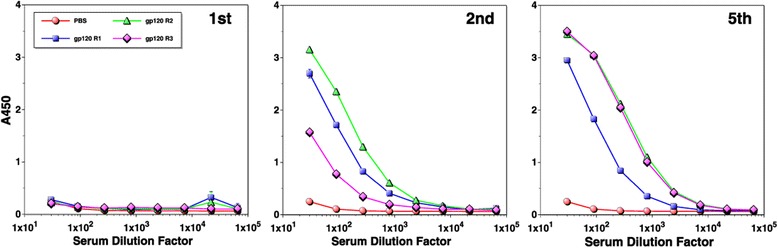
**Evaluation of antibody levels that cross-react with eOD-GT6.** ELISA was conducted with rabbit immune sera after the first, second, and fifth immunizations using eOD-GT6 as the coating antigen. Serum samples from a mock-immunized animal are indicated as “PBS”.

**Figure 6:**
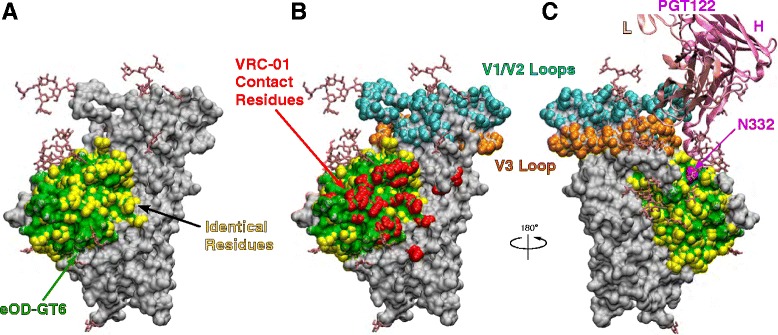
**Illustration of structural elements on gp120 and eOD-GT6. (A)** The crystal structure of SOSIP gp140 (accession code 4NCO) is used for the illustration. Gp120 is shown in grey, and regions corresponding to eOD-GT6 are shown in green. Amino acid residues identical between the MCON6 gp120 and eOD-GT6 are shown in yellow. **(B** **and C)** VRC01 contact residues are shown in red and V1/V2 and V3 loops are shown as a point of reference. PGT122 (an antibody related to PGT121 and PGT126) that was co-crystallized with SOSIP gp140 is shown with the critical N332 glycosylation site.

We synthesized nucleotide sequences encoding eOD-GT6 fused to Lumazine Synthase that self-assembles into 60-mer nanoparticles as previously reported [17], and constructed an expression plasmid. The protein was expressed in 293 F cells and purified from cell culture medium by a single-step affinity chromatography using Ni-NTA resin (Figure 4A). The protein was efficiently recognized by bnAbs VRC01 and NIH45-46, indicating structural integrity of the CD4BS (Figure 4B). ELISA was done with eOD-GT6 to assess the level of antibodies induced against MCON6 gp120 that could cross-react. As shown in Figure 5, no antibodies that bound eOD-GT6 were detected after the first immunization even at a 1:30 dilution. However, a moderate level of such antibodies was observed after the second immunization. The antibody levels increased only modestly even after five immunizations. Based on the data in Figure 3, these antibodies accounted for about 0.1% of the total antibodies directed against the outer domain. While cross-reactivity was very low, it should be noted that sequences of our gp120 immunogen and eOD-GT6 are only 74% identical. Furthermore, only a portion of the identical residues is exposed on the protein surface (Figure 6A).

### Induction of antibodies at or near the CD4 binding site

**Figure 7:**
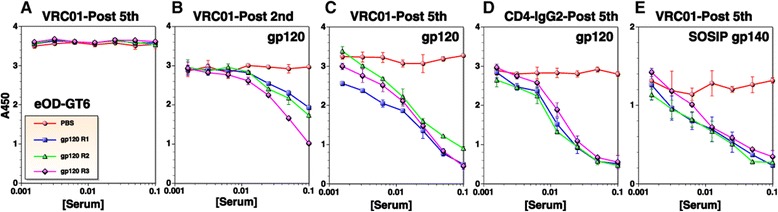
**Assessment of antibodies targeting near the CD4 binding site.** Binding of VRC01 **(A**, **B, C and** **E)** or CD4-IgG2 **(D)** to eOD-GT6 **(A)** gp120 **(B**, **C and** **D)** or SOSIP gp140 **(E)** was competed with sera from three immunized rabbits (gp120 R1-3) or mock-immunized rabbit (PBS). Immune sera after the second or fifth immunization were used.

As illustrated in Figure 6A and B, 88% of VRC01 contact residues on gp120 (indicated in red) are located on eOD-GT6 (green), and 74% of these residues are conserved between MCON6 gp120 and eOD-GT6 (yellow). Considering that there was a significant level of antibodies in anti-gp120 sera that bound eOD-GT6, albeit a small fraction of the total, we hypothesized that some of these antibodies could bind at or near the CD4BS. To test this hypothesis, we conducted antibody competition assays with VRC01 using eOD-GT6 and MCON6 gp120. When tested on eOD-GT6, antibodies from none of the rabbits could compete away VRC01 even at a 1:10 dilution of antisera collected after the fifth immunization (Figure 7A). Virtually identical results were obtained even when using ten-fold lower concentration of VRC01 (0.1 μg/ml; data not shown). In contrast, when tested on MCON6 gp120, approximately 50% of VRC01 could be competed at a 1:10 serum dilution collected after the second immunization (Figure 7B). Competing antibody levels increased modestly after the fifth immunization in all three animals (IC_50_ of approximately 1:40 dilution; Figure 7C). It is not clear why VRC01 could be competed away from MCON6 gp120, but not eOD-GT6. There are a few possibilities: (1) vaccine-induced antibodies that competed away VRC01 from gp120 are not “true” VRC01-class CD4BS-targeting antibodies and are unable to bind eOD-GT6, (2) the antibodies have higher affinity to gp120 than to eOD-GT6 since rabbits were immunized with the same gp120, and (3) VRC01 has higher affinity to eOD-GT6 than to gp120 ([17,18]; Figure 7). Nevertheless, the results indicated some antibodies targeted near the CD4BS close enough to compete with VRC01 binding to gp120.

To further characterize the antibodies that bind close to CD4BS, we performed a more direct competition assay with CD4-IgG2 with antisera collected after the fifth immunization. As shown in Figure 7D, gp120 antisera from all three rabbits inhibited binding of CD4-IgG2 to gp120. Considering a possibility that gp120 induced rabbit antibodies could compete with VRC01 or CD4-IgG2 binding to gp120, but not to trimeric envelope glycoprotein on the virion surface, we conducted a VRC01 competition assay with recently reported BG505 SOSIP gp140 [16,19]. As shown in Figure 7E, antisera blocked VRC01 binding to SOSIP gp140 with similar efficiency. Together, these data suggest that our vaccine regimen using MCON6 gp120 induced antibodies that bound in close proximity to CD4BS of gp120 and prevented binding to CD4.

### Induction of antibodies against epitopes targeted by other broadly neutralizing antibodies

**Figure 8:**
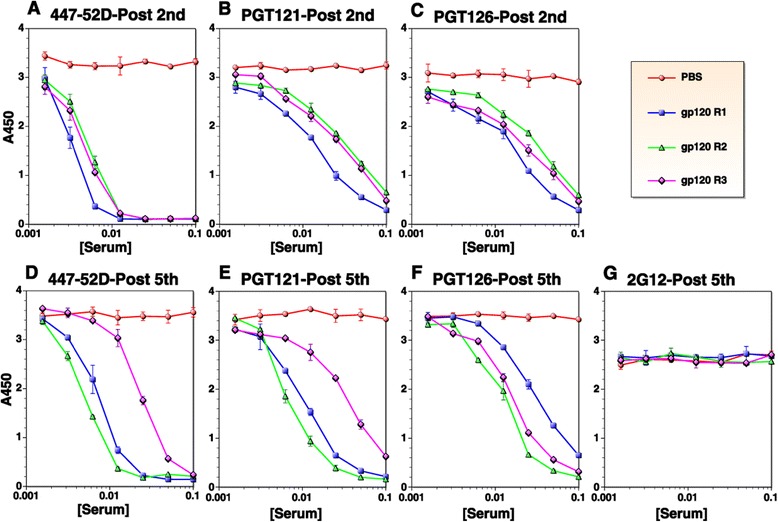
**Temporal analysis of serum antibodies targeting other known neutralizing epitopes.** Binding of 447-52D **(A and** **D)**, PGT 121 **(B and** **E)**, and PGT126 **(C and** **F)** to gp120 was competed with rabbit sera after the second or fifth immunizations. No competition was observed against 2G12 even after the fifth immunization **(G)**. Serum samples from a mock-immunized animal are indicated as “PBS”.

To further characterize antibodies induced by MCON6 gp120, we conducted competition assays using other nAbs: 447-52D that binds the tip of the V3 loop; PGT121 and PGT126 that bind a conserved region at the V3 loop stem near N332 glycosylation site; and 2G12, a unique antibody that binds an epitope comprised of a glycan cluster from C2, C3, V4 and C4 regions (Figure 8). As expected, 447-52D binding was efficiently blocked with antisera even after the second immunization (IC_50_ of 1:250) (Figure 8A and D). Both PGT121 and PGT126 were also competed (Figure 8B, C, E, and F), although not as efficiently as 447-52D. In general, antibodies that competed with these bnAbs increased after the fifth immunization, although there were exceptions (*viz.* rabbit #3). In contrast to these three nAbs, binding of 2G12 could not be blocked (Figure 8G), indicating the absence of antibodies that bound the epitope recognized by this unusual antibody.

## Discussion

For an AIDS vaccine to be effective, it must induce high levels of antibodies that can neutralize a majority of tier 2 HIV-1 isolates. Although our vaccine regimen using M group consensus sequence (MCON6) based gp120 induced potent and broad nAbs against tier 1 viruses, neutralizing activity against tier 2 viruses was weak and sporadic [14]. Despite the failure to induce potent bnAbs against tier 2 viruses, we believed it was important to fully characterize antibody responses to better understand why our immunogen failed and how we might be able to improve it in the future. Towards this goal, we conducted more detailed analyses of antibody responses in this study.

With respect to linear epitopes, the V3 loop was the major focus of the immune response from early on and it remained immunodominant throughout the course of immunization period (Figure 1). Based on an aggregate analysis of A450 values from ELISA, antibodies that bound V3 loop peptides accounted for ~18-20% of all antibodies that recognized linear peptides. Immunogenic linear epitopes were also identified in the C1 and C5 regions, antibodies to which would not exhibit neutralizing activity since they are not exposed on trimeric envelope spikes on virus particles. Based on subdomain ELISA analyses, strong antibody responses were also directed against the outer domain (Figure 3). Unfortunately, the proportion of antibodies directed against non-V3 loop that bound non-linear epitopes on the outer domain (*i.e*. those that cannot be detected by peptide ELISA) could not be readily determined with available reagents. To assess the level of antibodies directed against conserved core elements of the outer domain, we used eOD-GT6, a protein designed to bind VRC01-class bnAbs targeting the CD4BS and to their germline precursors [17]. About 0.1% of antibodies directed against the outer domain were able to bind eOD-GT6 after the second immunization. Although seemingly low, this cross-reactivity was considerable given that amino acid sequences of eOD-GT6 and MCON6 have only 74% identity.

Antibody competition assays revealed the presence of low, but significant levels of antibodies that bound at or near the vicinity of epitopes recognized by bnAbs VRC01, PGT121 and PGT126, at least close enough to sterically inhibit binding (Figures 7 and 8). Interestingly, the IC_50_ of antisera that blocked binding of VRC01 or CD4-IgG2 to gp120 (Figure 7C and D) corresponded closely to the mean ID_50_ of neutralizing activity against tier 2 viruses assayed in A3R5 cells (~1:90) [14]. Whether these antibodies that compete with VRC01 or CD4-IgG2 are indeed responsible for the weak neutralization of Tier 2 viruses is not yet known and will require additional studies. The induction of these antibodies was slower and at much lower levels compared to antibodies that competed with the anti-V3 loop nAb 447-52D, consistent with linear epitope mapping data (Figure 1). One interesting observation is that while competing activity against 447-52D remain unchanged or even decreased from the second to the fifth immunization (Figure 8A and D, respectively), activity against VRC01 (Figure 7B and C), PGT121 (Figure 8B and E) and PGT 126 (Figure 8C and F) generally increased. The increased competing activity could be due to actual increase in antibody quantity, increased affinity between the antibodies and target epitopes on gp120, or a combination of both. Regardless, the data suggested that regions near the bnAb epitopes were continuously being probed by the immune system following each immunization. In this regard, additional characterization of the evolution and affinity maturation of these antibodies during the course of immunization may provide better insights as to how to modify vaccine strategies to optimize bnAb induction. This would require analyses at both clonal (*e.g.* mAbs) and populational levels (*i.e*. through deep sequencing of antibody genes).

Not all vaccine-induced antibodies that compete with known bnAbs are expected to exhibit neutralizing activity. There could be antibodies that block binding of bnAbs to gp120, but unable to block CD4 binding. Some antibodies might block binding of bnAbs to gp120, but not to a trimeric envelope complex, due to more restricted angles of approach on the trimer. Similarly, some of the conventional anti-V3 loop-specific antibodies could interfere with PGT121/126 bnAbs. Steric hindrance by antibodies simply cannot guarantee targeting of the same epitopes recognized by bnAbs and subsequent inhibition of critical envelope functions (*e.g.* CD4 or coreceptor binding, or membrane fusion). Notwithstanding these caveats, the fact that our vaccine-induced antibodies could inhibit binding of CD4-IgG2 to gp120 and binding of VRC01 to SOSIP gp140 raises the likelihood that some antibodies might exhibit neutralizing activity. Thus, despite low antibody levels, our results are significant, especially when considering that VRC01, PGT121 and PGT126 are some of the most potent bnAbs against HIV-1.

Despite some success in inducing antibodies that target near the epitopes recognized by known bnAbs (*e.g.* CD4BS), much more improvement is needed to induce stronger antibody responses against them. As shown in Figure 7A, the antibodies failed to block binding of VRC01 to eOD-GT6 even though they inhibited binding to gp120 and SOSIP gp140, suggesting that the antibodies were not targeting the CD4BS precisely and/or that the affinity is not high enough in the context of eOD-GT6. Not only better immunogens, but better immunization strategies and vaccine formulations are likely needed to coax the humoral immune system to focus on bnAb-specific epitopes like CD4BS while minimizing responses to decoy epitopes like the V3 loop [20-23]; also see [24] and references therein. First of all, immunogens used must have structural integrity of known bnAb epitopes. Secondly, immunogens must also engage germline B cell receptor (BCR) precursors that give rise to bnAbs, an event that initiates B cell activation, BCR diversification through somatic hypermutation, and antibody affinity maturation. Thirdly, the immunogens must minimize inducing strain-specific- or non-neutralizing antibodies. HIV-1 bnAbs require a large number of somatic mutations [25,26]. As such, vaccine formulations and regimens that allow proper and efficient evolution of germline BCRs into mature bnAbs would be critical.

**Figure 9:**
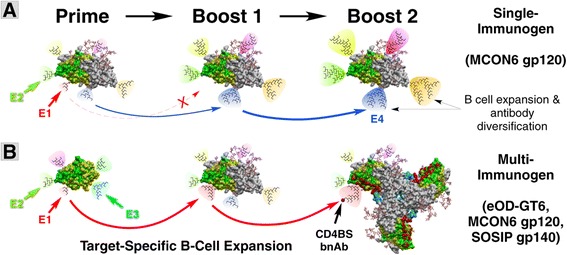
**Comparison of hypothetical antibody responses against single versus multi-immunogen vaccine regimens.** Two vaccine regimens are compared: **(A)** single immunogen (*e.g.* gp120) and **(B)** multiple immunogens (*e.g.* eOD-GT6, gp120 and SOSIP gp140). The portion of the protein that corresponds to eOD-GT6 is shown in green. Amino acid residues identical among all three immuogens are indicated in yellow, and those that contact VRC01 are identified in red. The extent of hypothetical B cell expansion and antibody diversification is illustrated by different sizes of the cone and phylogenetic trees. Epitopes targeted by undesirable antibodies (strain-specific or non-neutralizing) are exemplified as epitopes E2 or E4. An immunorecessive epitope (*e.g.* CD4BS) targeted by VRC01-class antibodies is exemplified as E1 epitope.

To date, no single immunogen has been generated that meets all of the criteria described above. One possible strategy to overcome this difficulty is to utilize multiple heterologous immunogens as exemplified in Figure 9. In contrast to a traditional vaccine regimen where the same immunogen is used to “prime” and “boost” immune responses (*e.g.* gp120, Figure 9A), a heterologous multi-immunogen vaccine regimen would use multiple immunogens that are related, yet different in not only overall structure, but also in amino acid sequence (*e.g.* eOD-GT6, MCON6 gp120, and BG505 SOSIP gp140 trimer; Figure 9B). When using a single immunogen, strain-specific- or non-neutralizing epitopes could be immunodominant (*e.g.* V3 loop or C1 and C5 regions in the inner domain; epitopes E2 and E4 in Figure 9A). Antibody responses against weakly immunogenic epitopes (*e.g.* CD4BS; epitope E1 in Figure 9A) could decline with each successive immunization. In contrast, when using multiple heterologous immunogens, the immune system will be forced to identify a “structural consensus” among all immunogens. For example, antibodies against the E3 epitope generated by priming with eOD-GT6 would not be further amplified because that epitope is missing on gp120 (Figure 9B). Similarly, antibodies against epitopes such as E2 on eOD-GT6 would not be amplified because the amino acid sequence is different on gp120 and SOSIP gp140. Consequently, antibodies targeting epitope E1 could expand with successive boosting with gp120 and SOSIP gp140 despite being immunorecessive. We hypothesize that successive use of these immunogens could guide targeted epitope-directed antibody evolution through what may be considered as “antigenic synergy”. In this example, eOD-GT6 activates germline B cells targeting the CD4BS without stimulating those that target decoy epitopes; gp120 would be an intermediary that further allows expansion of antibodies that target CD4BS; finally, SOSIP gp140 would then refine and select antibodies that could bind to native trimeric envelope spikes on virus particles. While our current vaccine regimen with MCON6 gp120 failed to induce antibodies that could compete with VRC01 binding to eOD-GT6, we predict that a multi-immunogen vaccine strategy shown in Figure 9B would be much more efficient in inducing high titers of such antibodies.

## Conclusions

In this study, a detailed characterization of antibody responses against MCON6 gp120 in rabbits was carried out by overlapping peptide ELISA, subdomain target analyses and competition assays with known bnAbs. As expected, the V3 loop was most immunogenic. About 0.1% of the antibodies bound eOD-GT6, a protein that was designed to bind bnAbs targeting the CD4BS and their germline precursors. Significant levels of antibodies that bound at or near the epitopes targeted by bnAbs VRC01, PGT121 and PGT126 were detected. Despite some success, additional work will be needed to induce bnAbs, especially in developing new vaccine strategies that incorporate multiple immunogens to focus antibody responses to critical neutralizing epitopes and to better guide antibody maturation.

## Methods

### Construction, expression and purification of eOD-GT6

The amino acid sequence of eOD-GT6 fused to Lumazine Synthase was previously reported [17]. Human codon-optimized gene sequence was synthesized commercially (Life Technologies), with restriction sites AgeI and KpnI introduced at 5’ and 3’ ends, respectively. The synthesized gene was cloned into pHLsec mammalian expression vector (kindly provided by Dr. E. Yvonne Jones, [27]) through corresponding restriction enzyme sites. The protein was expressed following transfection of the resulting plasmid into FreeStyle 293-F cells using 293fectin (Invitrogen, Life Technologies) as per manufacture’s protocol. Four days post-transfection, the culture medium was harvested and purified using Ni-NTA agarose beads. The eluted protein was concentrated with an Amicon Ultraconcentrator (Millpore) and stored at −80°C until use. Protein purity was assessed by silver stain, and its structural integrity was confirmed by ELISA using VRC01 [26] and NIH45-46 [28].

### ELISA with overlapping peptides or protein

ELISAs were performed as previously described [14] with some modifications. For peptide ELISA, peptides were coated onto 96-well Nunc-Immuno Plates overnight at 4°C at 20 pmol per well using an antigen coating buffer (15 mM Na_2_CO_3_, 35 mM NaHCO_3_, 3 mM NaN_3_, pH 9.6). Uncoated surfaces were blocked with 200 μl of a blocking buffer (PBS, pH 7.4, containing 2.5% skim milk and 25% Fetal Bovine Serum) for 1 h at 37°C. Wells were subsequently washed five times with a wash buffer (PBS containing 0.1% Tween 20) using a Biotek automated plate washer. For peptide ELISA (Figure 1), rabbit sera were diluted 1:100 in blocking buffer (1:50 for post-second immunization samples), and 100 μl was added to each well. Plates were then incubated for 2 h at 37°C. Wells were washed ten times, and plates were further incubated for 1 h at 37°C with secondary antibody (horseradish peroxidase (HRP)-conjugated goat anti-rabbit IgG (H + L) (Pierce) at 1:3000 fold dilution in blocking buffer. Wells were washed again ten times, and the HRP reaction was initiated by adding 100 μl TMB HRP-substrate (Bio-Rad). The reaction was stopped after 10 min by adding 50 μl of 2 N H_2_SO_4_. Plates were read on a microplate reader (Versamax by Molecular Devices) at 450 nm. All assays were done in duplicate.

The 15-mer overlapping peptide set for gp120 based on the M group consensus sequence (CON-S) was obtained from the NIH AIDS Reagent Program (Cat# 9487). V1, V2, V4 and V5 peptides based on the MCON6 sequence (H-VRNVSSNGTETDNEE-OH, H-DKNSSEISGKNSSEY-OH, H-MFNGTYMFNGTKDNSE-OH and H-GNNSNKNKTETFRPG-OH, respectively) were synthesized commercially (CHI Scientific, Maynard, MA). For whole protein ELISA, the indicated purified immunogens were coated as described at 30 ng per well. All sera and antibodies were serially diluted in blocking buffer as indicated in figures, and 100 μl was added to each well. Secondary antibodies were HRP-conjugated goat anti-rabbit IgG (H + L) (Pierce) for Figures 3 and 5 and HRP-conjugated goat anti-human IgG (H + L) (Pierce) for Figure 4.

### Antibody competition assays

Competition assays (Figures 7 and 8) were performed by modifying the described ELISA protocol. Coating antigens used were eOD-GT6, MCON6 gp120 or BG505 SOSIP gp140. The gp120 protein was expressed and purified from S2 cells, similar to our previous study [14]. BG505 SOSIP gp140 (kindly provided by Dr. John P. Moore, [16,19]) was expressed from FreeStyle 293 F cells (Invitrogen, Life Technologies) and purified using lectin agarose beads (Cat# AL-1243; Vector Laboratories) as per the manufacturer’s protocol. Antigens were coated at 30 ng per well. Briefly, serum was diluted 1:5 in an alternate blocking buffer (5% calf serum and 2.5% milk in PBS). This initial dilution was then subjected to two-fold serial dilutions. Monoclonal antibodies were diluted to a concentration of 2 μg/ml in blocking buffer. 50 μl of the diluted antibody was added to each well along with 50 μl of the serially diluted sera. Hence the final monoclonal antibody concentration during the assay was 1 μg/ml, and the starting serum dilution was 1:10. The plate was briefly mixed on a plate shaker and then incubated for 90 min. Fc specific, HRP conjugated rabbit anti-human antibody (Pierce) was diluted 1:3000 in the blocking buffer as a secondary antibody. Detection was performed as described for the standard ELISA. Antibodies used for competition included VRC01 [26], CD4-IgG2 [29], 447-52D [30-35], PGT121 [36,37], PGT126 [36], and 2G12 [38-42].
